# Therapeutic effects of sialendoscopy for diagnosis and treatment of hyposalivation patients: a retrospective study

**DOI:** 10.1186/s40902-022-00360-8

**Published:** 2022-10-24

**Authors:** Seung-Jun Lee, Euy-Hyun Kim, Sung-Jae Lee, Young-Joon Chun, In-Seok Song, Sang-Ho Jun

**Affiliations:** Department of Oral and Maxillofacial Surgery, Korea University Anam Hospital, 73, Goryeodae-ro, Seongbuk-gu, Seoul, Republic of Korea

**Keywords:** Hyposalivation, Sialendoscopy, Scintigraphy, Salivary gland, Sjogren’s disease

## Abstract

**Background:**

Hyposalivation is disease with multiple symptoms. This disease is hard to be diagnosed and to be treated, and there are not enough clinical protocols to cure the disease. In this study, we propose our own treatment protocols which aim not only to cure the disease but also to care for the disease-related symptoms.

**Methods:**

At the 1st visit, we collect patient-related information. This procedure includes an intraoral exam, patient history taking, VAS value and unstimulated whole saliva (UWS) measurement, and salivary buffer test. Following the interview and oral examination, objective results are obtained by radiological image, CT, and sialoscintigraphy. At the 2nd visit, we analyze radiographic images including neck CT and salivary scintigraphy. These images can allow accurate diagnosis and help the patients to better understand the current condition. Depending on the severity of symptoms and patient’s discomfort, we try a surgical approach at the 3rd visit, sialendoscopy.

**Results:**

With treatment, we can manage the discomfort of patients in daily life. The VAS value of hyposalivation patients dropped gradually with the trial of sialendoscopy. In the case of Sjogren’s syndrome patients, the treatment efficacy has been decreased with low reactivity of treatment. The true meaning of this treatment is in not only curing the disease, but also caring for the disrupted patients. Overall, the amount of UWS increased with the progress after the procedure. Especially in the lower UWS at the 1st visit, there was a more significant increase after the procedure.

**Conclusion:**

Although many factors that cause hyposalivation have not been identified, the efficacy of sialendoscopy to relieve discomfort in hyposalivation patients has been observed. However, treatment was more difficult and complicated in the group of patients with systemic disease. This study will not only present a treatment protocol for hyposalivation patients, but also consider methods for diagnosing more precisely and improving treatment efficacy. Hyposalivation is a curable and manageable disease in some cases, so interpretation between the clinician and the patient is important.

## Background

Hyposalivation is frequently found in age group more than 50s. Lots of factors affect this disease, which are aging, systemic diseases, medications, radiation, environmental factors, malnutrition, auto-immune disease, and so on [1]. A typical symptom of this disease is dryness of the oral region. Additionally, fissured tongue and insomnia can occur among hyposalivation patients [2].

However, this disease is difficult to be diagnosed. That is, symptoms are regarded as somewhat trivial. But it makes further complications, which are hard to diagnose and to be treated. And if there were additional general diseases existing, the treatment becomes more complicated. Much of drugs can aggravate the salivation of patients, such as anti-depressants and steroids. Climacteric drugs including hormones can affect salivation either. If a clinician wants to get closer to a genuine remedy of hyposalivation, a comprehensive approach is needed including general disease, drug history, host factors, and so on.

Conventional treatment is somewhat ambiguous and allopathic. There are no sufficient treatment protocols concerning hyposalivation. Because this disease is multifactorial, the therapeutic approach is complicated depending on the cause and symptom of the patient.

In this study, we propose a new treatment protocol for hyposalivation patients in Korea University Medicine OMS department. We try to approach diagnosing with radiographic and clinical methods, enhancing the salivation, and treating other medical diseases. This protocol also includes active treatment, such as several diagnostic tools and sialendoscopy [3]. At the same time, medication is going side by side. Our clinical goals are not only treating the hyposalivation, but also caring for the symptoms. If a patient does not give up, we give patients constant care of hyposalivation-related symptoms and complications.

## Methods

We collected data of 154 patients who were given sialendoscopy, from 2018 to 2021.12 in Korea University Medicine OMS department. Patients had symptoms of dryness of the oral cavity. We compared results before and after the treatment to analyze the efficacy of the treatment.

**Fig. 1:**
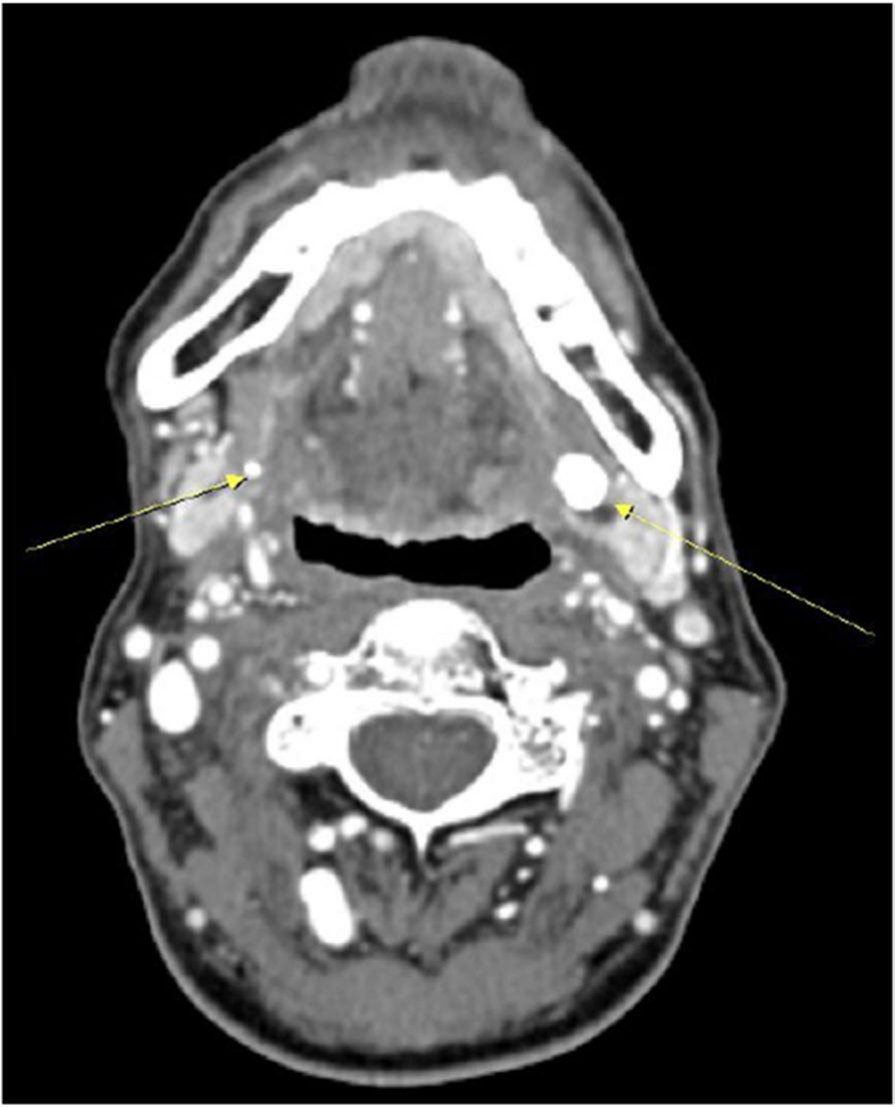
Neck CT (enhanced) image with sialolithiasis

**Fig. 2:**
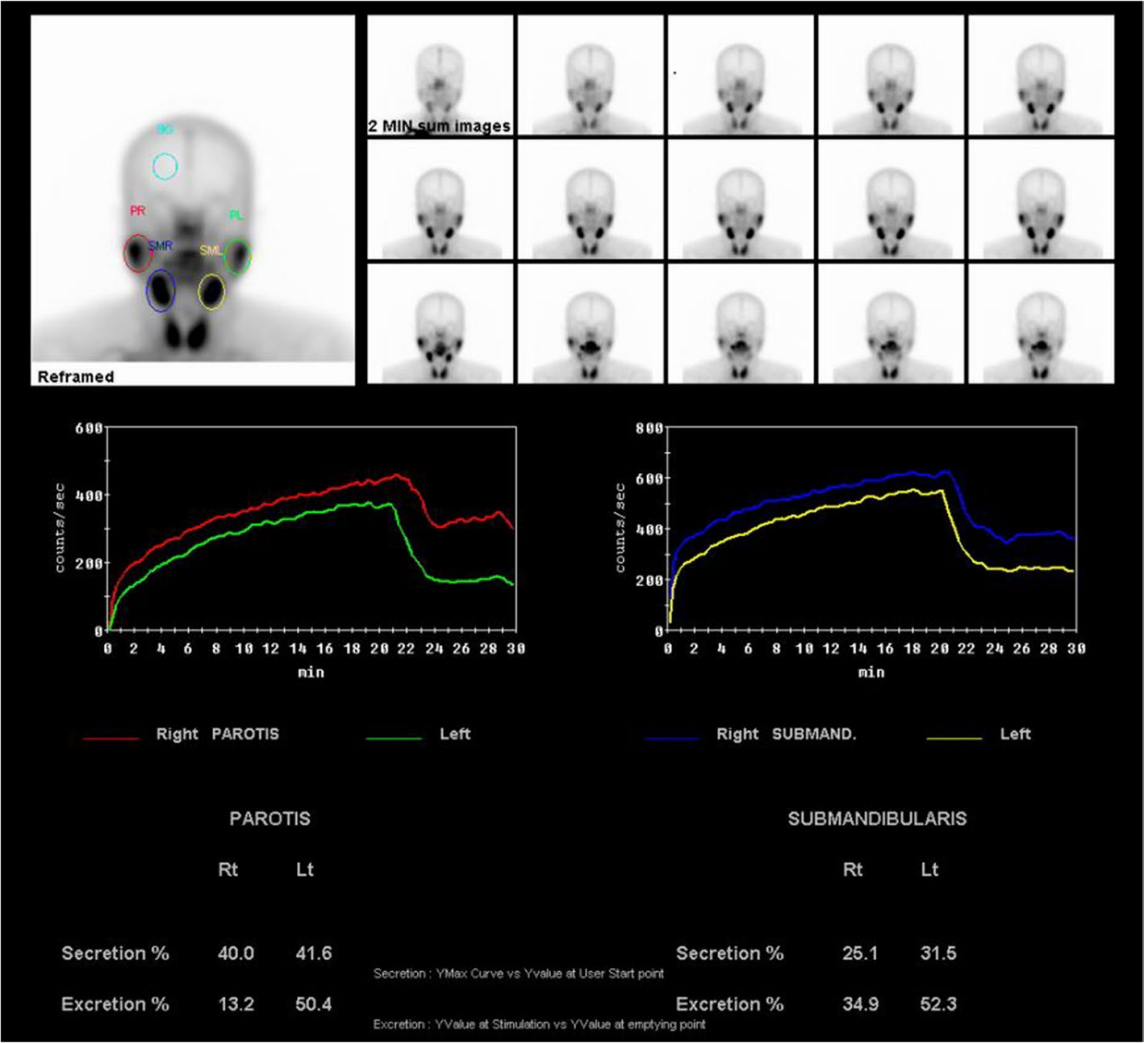
Salivary scintigraphy image with decreased salivary flow

**Fig. 3:**
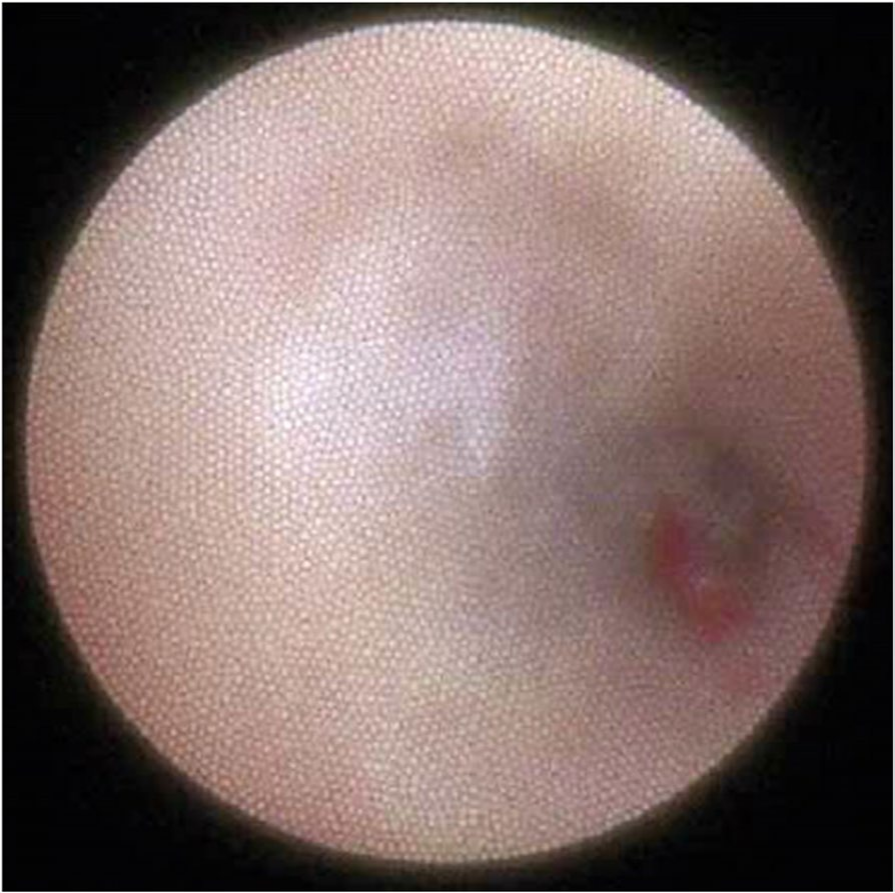
Wharton’s ductal status recorded by sialendoscopy. An obvious inflammatory state with mucous plugs can be detected

We subdivided the data of 154 patients, and among them, 50 patients who measured both the pH meter and unstimulated whole saliva (UWS) were detailed from 2020.6 to 2021.12 in Korea University Medicine OMS department. Patients were classified based on the amount of UWS secretion at the time of 1st visit, and the amount of UWS and changes in pH were examined before and after the procedure. The classification was divided into 5 groups with 0.5-ml increments ranging from 0 to 2ml and over 2ml of UWS. Below, we described the treatment protocols of our clinic (Figs. 1, 2, and 3).

Our treatment protocol for systematic treatment according to patient’s comorbidity is as follows. The patient will carefully confirm the medical history and hyposalivation symptoms performed at the 1st visit through interviews and obtain objective results through image taking. It can acquire unstimulated whole saliva and indirectly cause patient symptoms. In order to deeply observe the patient’s discomfort, a questionnaire using the VAS score is conducted at the time of the interview [4].

**Fig. 4:**
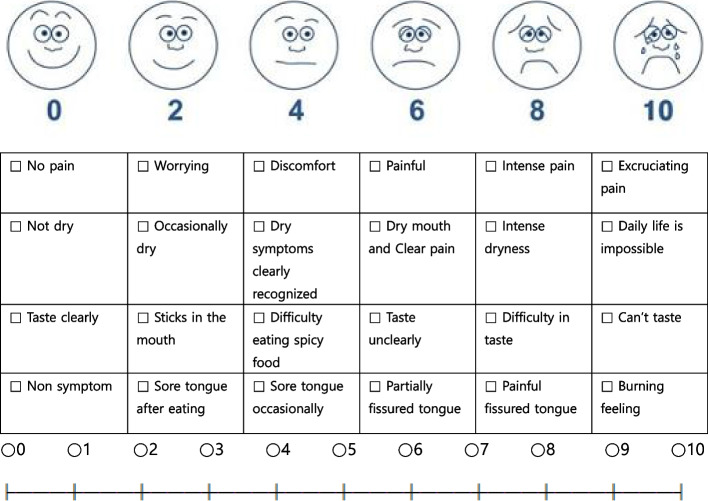
A questionnaire conducted for patients based on the patient’s discomfort

The questionnaire was divided into four categories, each consisting of overall discomfort, dry mouth, taste, and tongue symptoms (Fig. 4).

1. Discomfort (0: no pain, 2: worrying, 4: discomfort, 6: painful, 8: intense pain, 10: excruciating pain)
2. Dryness (0: not dry, 2: occasionally dry, 4: dry symptoms clearly recognized, 6: dry mouth and clear pain, 8: intense dryness, 10: daily life is impossible)
3. Taste (0: taste clearly, 2: sticks in the mouth, 4: difficulty eating spicy food, 6: taste unclearly, 8: difficulty in taste, 10: cannot taste)
4. Tongue (0: non-symptoms, 2: sore tongue after eating, 4: sore tongue occasionally, 6: partially fissured tongue, 8: painful fissured tongue, 10: burning feeling)

This survey is advantageous for asking patients about their discomfort in detail. The visual analog scale (VAS) is a tool widely used to measure pain. In this study, the VAS index is also used to measure the patient’s discomfort, and VAS is defined as “Indication of his/her perceived pain intensity along a 100mm horizontal line.” The state of salivary glands and ducts can be confirmed from the enhanced CT image, and the dynamic state of salivary secretion and excretion can be confirmed through sialoscintigraphy.

The 2nd visit provides the patient with objective symptoms and treatment directions based on the examination results. The degree of atrophy of the patient’s major salivary gland and duct can be described as an objective value. Correlation with the affected systemic medical history can also be inferred and methods for alleviating the patient’s symptoms will be described.

Sialoendoscopy will be performed at the 3rd visit. It is performed according to the patient’s symptoms and is very useful not only for treatment but also for diagnosis. In treatment, sialendoscopy is attempted by dilating the ducts, rinsing the irritant substances such as mucous plugs, and flushing the drug to the inflamed area. After that, a periodic follow-up is usually performed 3 days, 1 month, 3 months, and 6 months after the treatment.

The efficacy of sialendoscopy was analyzed with VAS, UWS, and pH measured during all processes for each patient. As a statistical method, the ANOVA test was performed and *p*=.05 significance was used. Statistical methods Scheffe and Dunnett T3 were used as a post hoc test to analyze the specific differences between the groups after the ANOVA test.

## Results

**Fig. 5:**
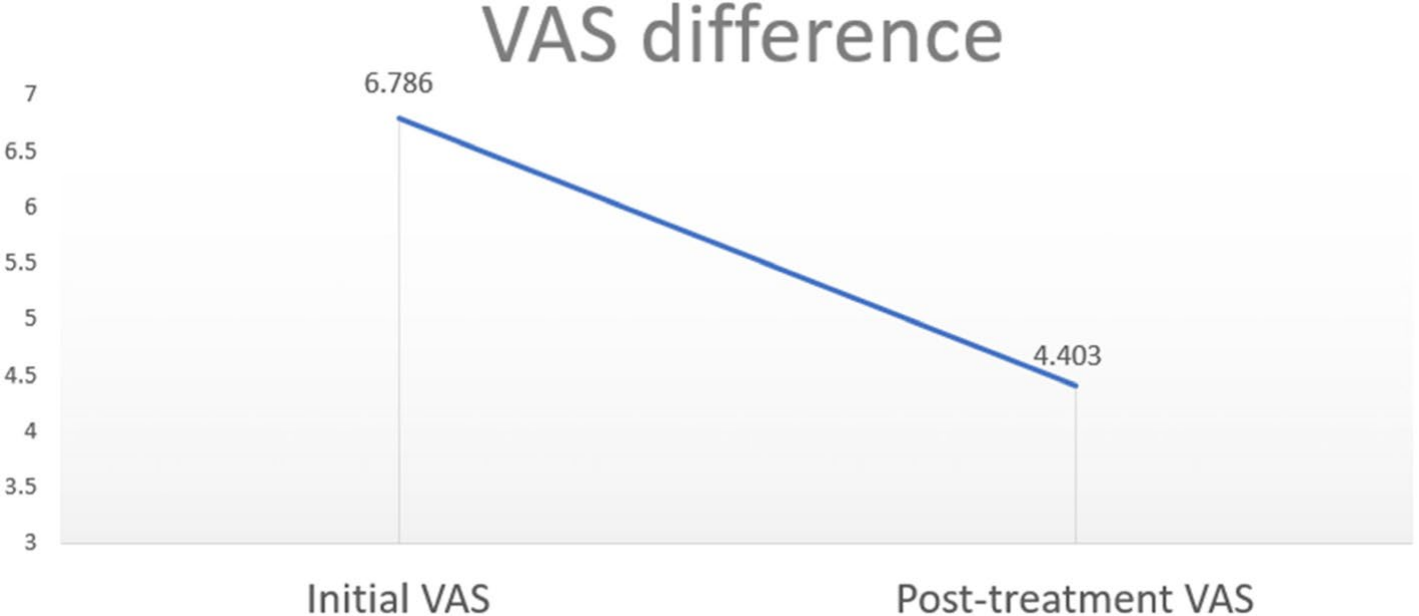
After the treatment, the VAS value of all 154 patients decreased

**Fig. 6:**
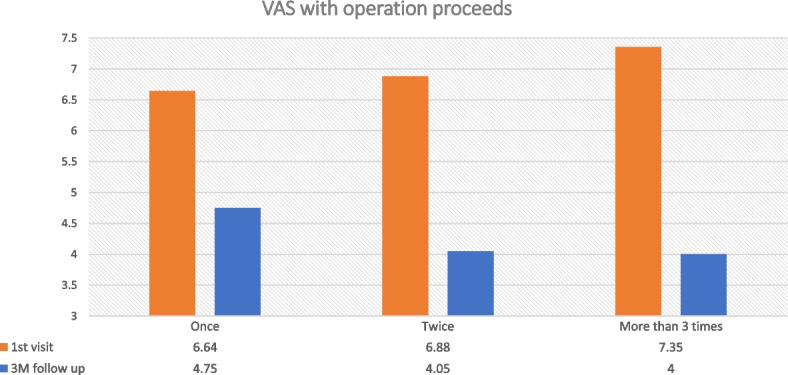
The VAS at the 1st visit and the VAS after the treatment were compared according to the number of sialendoscopy

Forty-nine males and 105 females were included in the study. In the first visit, the average VAS value of patients was 6.785, and it gradually dropped to 4.392 as treatment progressed (Fig. 5). And the data showed statistically significant (*p*< .001). The decreasing trend of the VAS index of all patients was confirmed, and to evaluate the efficacy of sialendoscopy in detail, the change in VAS according to the number of treatments in the group of patients who underwent more than 3 procedures was examined. In addition, we divided the groups based on the number of treatments and observed the change in VAS according to the number of treatments. In detail, efficacy according to the number of sialendoscopy was investigated by grouping patients who had undergone the procedure only once, 2, and 3 times or more [5]. In the group of patients who underwent the procedure only once, there was a decreasing trend from VAS 6.64 at the 1st visit to 4.75. In the group of 2 times procedure, the VAS index decreased from 6.88 at the 1st visit to 4.05. In the group of 3 times procedure, the VAS index decreased from 7.35 at the 1st visit to 4.00 (Fig. 6). Regardless of the number of treatments, the difference between the VAS at the 1st visit and the VAS after sialendoscopy was statistically significant (*p*<0.001). In addition, in order to compare the efficacy of sialendoscopy according to the number of procedures, when the difference in VAS between groups before and after the treatment was statistically analyzed using Dunnett T3 (Table 1), the difference between the group that had the treatment once and the group that had the treatment 2 times was found to be statistically significant (*p*=.002). However, the difference between the group that had the treatment 2 times and the group that had the treatment 3 times was not statistically significant (*p*= .364).

**Table 1:** Statistical analysis of the VAS difference before and after the sialendoscopy depending on the number of treatments

Mean value of VAS difference, Dunnett T3						
The number of Tx(I)	The number of Tx(J)	Mean	SD	*P* value	95% CI	
					Lower limit	Upper limit
1	2	1.51	0.43	0.002*	0.45	2.56
	3	2.53	0.61	<.001*	0.99	4.08
2	1	-1.51	0.43	0.002*	-2.56	-0.45
	3	1.02	0.68	0.364	-0.67	2.72
3	1	-2.53	0.61	<.001*	-4.08	-0.99
	2	-1.02	0.68	0.364	-2.72	0.67

*Data sharing the symbol (*) differ significantly (*P*<0.05)

**Fig. 7:**
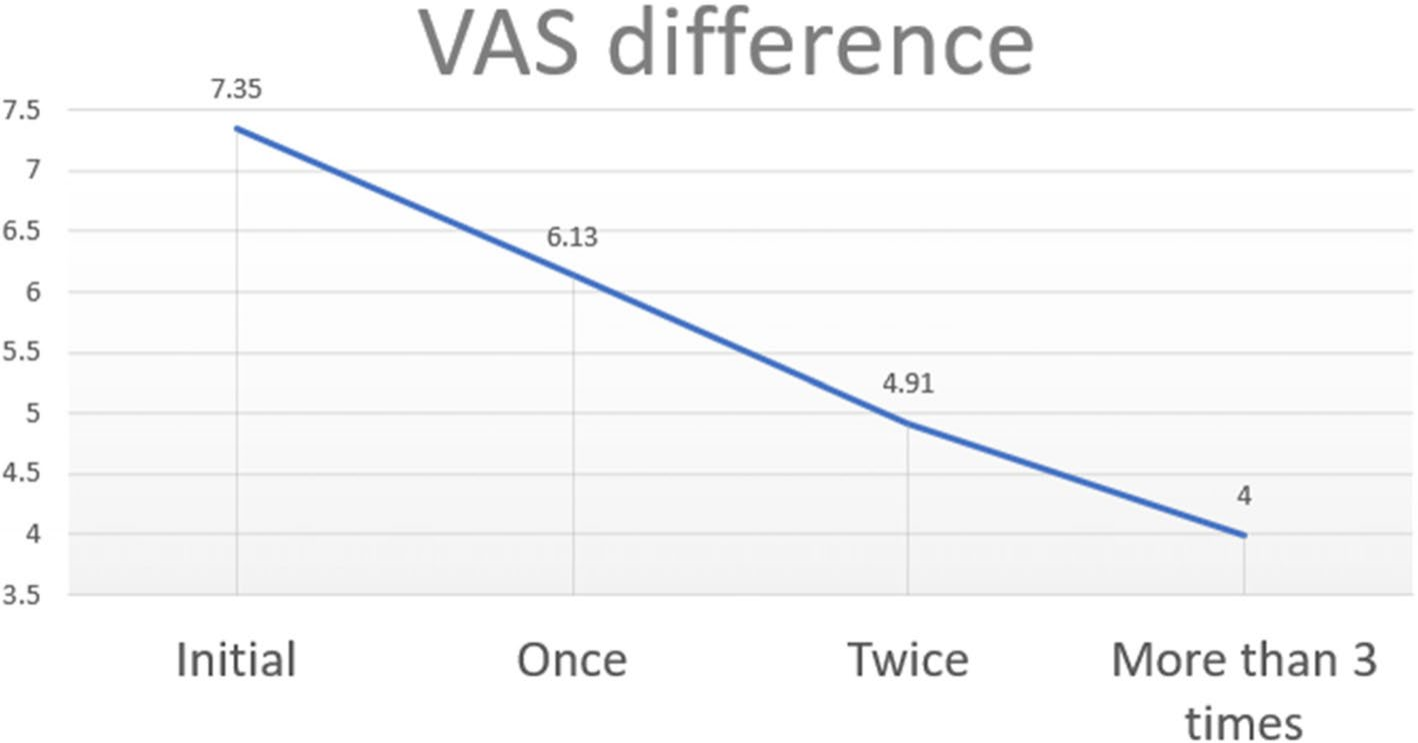
The graph shows that VAS decreases depending on the number of treatments in the group of patients who underwent sialendoscopy three or more times

**Fig. 8:**
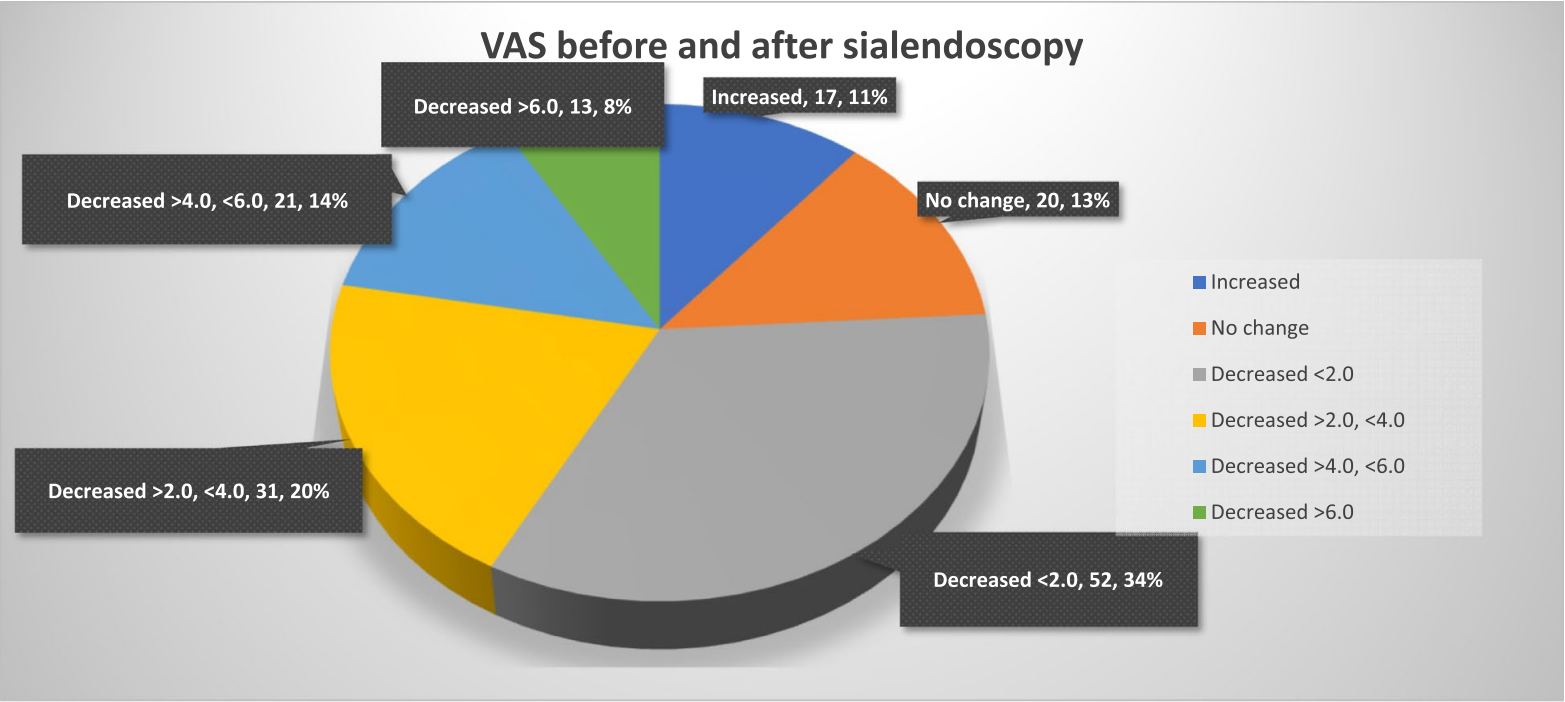
The graph shows groups according to the amount of change in VAS before and after sialendoscopy. More than half of the patients had VAS decreased from 0 to 4 after treatment

On the other hand, in the group of patients who underwent 3 or more procedures, the VAS index decreased according to the number of treatments. One month after the 1st sialendoscopy, the average VAS was 6.13. One month after the 2nd sialendoscopy, the average VAS was 4.91. More than 3rd trial of sialendoscopy, the average VAS dropped to 4.00 (*p*< .001) (Fig. 7). After the treatment, 117/154 patients had improvement in their symptoms (Fig. 8). Fifty-two of 154 patients’ VAS value were decreased less than 2.0, which was the biggest proportion in this study. The most successful group was the group that more than 6.0 VAS value was decreased, 13/154 patients. In contrast, 20/154 patients have no change in their symptoms. And other 17/154 patients have gotten even worse than before the treatment. After the treatment, 9/154 patients were completely cured, resulting in a VAS value of 0. Sixteen of 154 of them were having a resulting VAS value of 1, and 16/154 of them were having a resulting VAS value of 2. And we regrouped them and named as successful care groups.

**Fig. 9:**
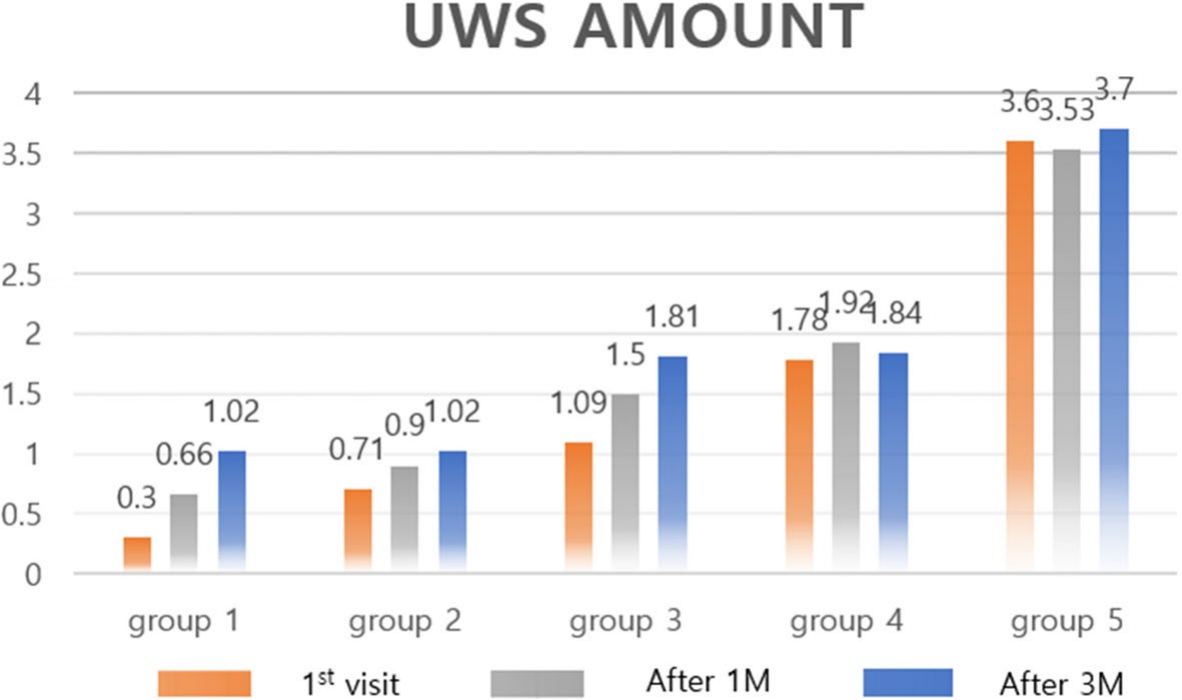
Changes in UWS for 3 months of follow-up visit after sialendoscopy in the group according to UWS amount at the 1st visit

The additional results were analyzed for 50 of 154 patients who measured both the pH meter and unstimulated whole saliva. The results of follow-up 3 months after the procedure were analyzed [6]. The changes after the 1st visit, 1 month, and 3 months after the procedure were shown in a graph (Fig. 9). In groups 1, 2, and 3, a gradual increase in the amount of UWS was observed according to the progress. In group 1, the average UWS of patients (*n*=7) was 0.3ml/10min at the 1st visit. And 1 month after the procedure, it gradually increased to 0.66ml/10min. Three months after the procedure, it increased to 1.02ml/10min. In group 2, the average UWS of patients (*n*=14) increased to 0.71ml/10min at the 1st visit, to 0.9ml/10min after 1 month, and to 1.02ml/10min after 3 months. In group 3, the average UWS of patients (*n*=8) increased to 1.09ml/10min at the 1st visit, to 1.5ml/10min after 1 month, and to 1.81ml/10min after 3 months. In groups 4 (*n*=5) and 5 (*n*=16), the amount of UWS remained similar.

**Fig. 10:**
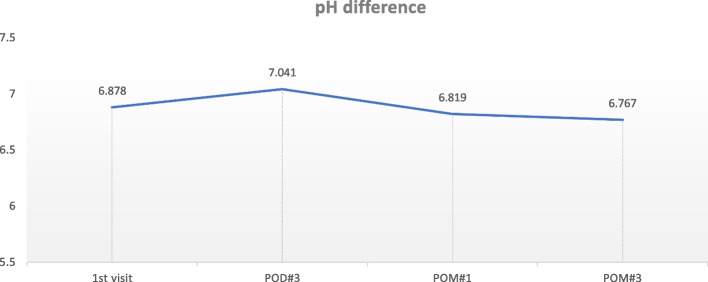
Changes in pH for 3 months of follow-up visit after sialendoscopy

The pH before and after the sialendoscopy was all within the normal range which is 6.3~7.6, and it is considered that there was no significant change according to the progress after the sialendoscopy (Fig. 10).

## Discussion

In general, hyposalivation is diagnosed when the amount of unstimulated whole saliva is less than 0.1ml per minute [7]. Therefore, through more efficient diagnosis and treatment, the UWS is increased to contribute to the reduction of patient discomfort. First, the severity of chronic sialoadenitis was assumed based on the amount of salivation and grouped by the amount of UWS at the 1st visit. The increase in UWS ranged from 3 to 240% (Fig. 9). The lower UWS at the 1st visit, the more significant increase after the procedure. In group 1, a relevant increase in UWS of 120% at the visit after 1 month was observed. At the 3 months after the procedure, it showed an increase by 240%. Similar to group 1, group 2 showed a relevant increase of 26% after 1 month and 43% after 3 months, and group 3 showed a relevant increase of 37% after 1 month and 66% after 3 months. In the group with higher UWS, the change after the procedure was not significant. It showed an increase of 2~3% after the procedure. The smaller the amount of UWS at the 1st visit, the greater the increase in UWS after sialendoscopy. It is thought that significant results can be obtained by treating more patients and collecting data in the future.

Medical comorbidity can deteriorate the results of treatment, such as thyroid disease with radioactive treatment [8], mental disorders, and autoimmune diseases like Sjogren’s disease [9]. These diseases originally aggravate the function of the parenchyma of the salivary gland, which leads to limited healing potential. These patients needed additional treatment related to their own medical department with our care [10].

Sialendoscopy is conducted worldwide with its efficacy [11]. It can enhance saliva secretion and the function of the salivary gland. With direct irrigation with saline and diluted dexamethasone solution, we can alleviate inflammation of salivary ducts and wash out remnants in ducts such as mucous plugs by flushing the solution.

**Fig. 11:**
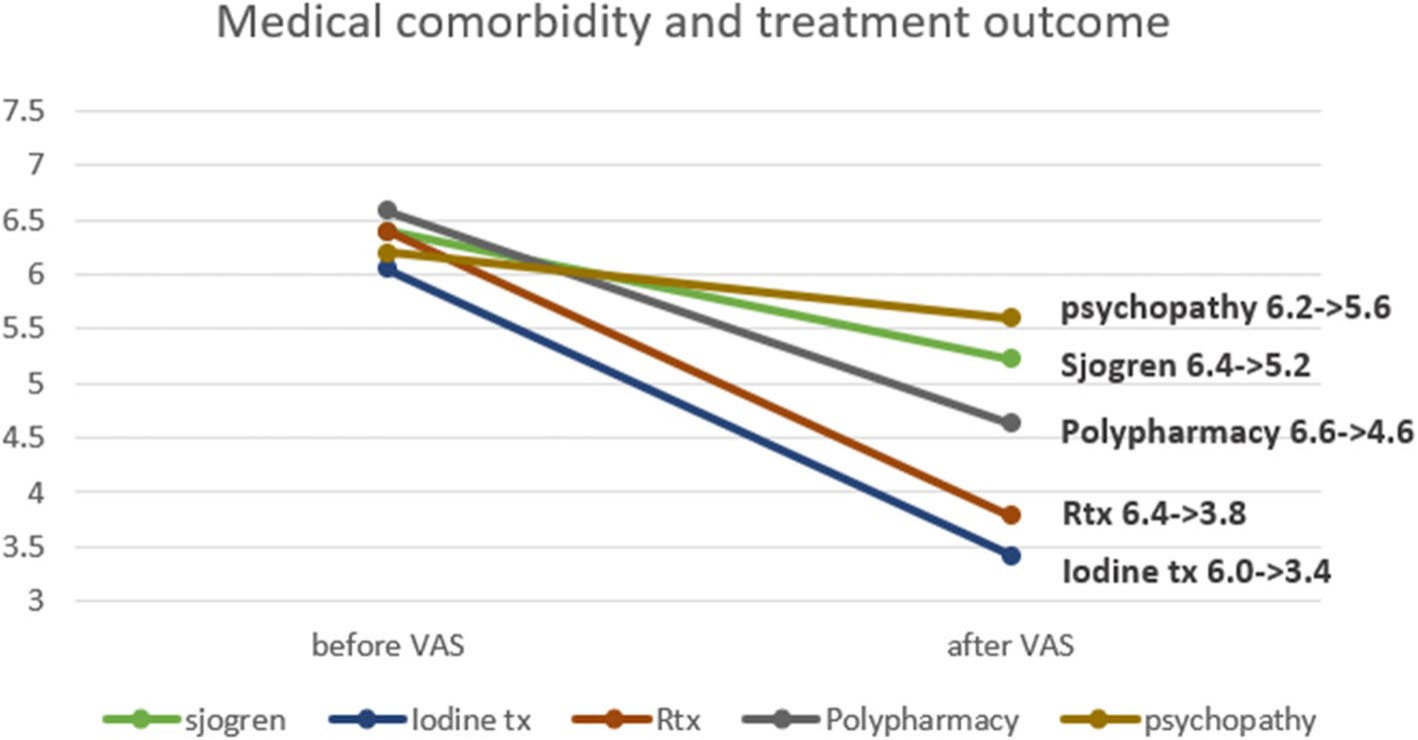
Correlation of VAS scale and medical comorbidity. Reactivity of treatment differs from the type of medical comorbidity of patients

Medical comorbidity is closely related with the severity of the disease and the treatment outcome of patients (Fig. 11). In the case of psychopathy patients, treatment efficacy had been worst among the patient groups. Patients with a history of oro-maxillofacial radiation therapy or iodine therapy are generally classified as intractable patients [12]. In our data, the VAS value of patients treated with radiation dropped from 6.4 to 3.8 and the VAS value of patients with iodine therapy dropped from 6.0 to 3.4, which were the best treatment efficacy. Because of the low therapeutic potential of the Sjogren’s syndrome patient group, clinicians should consider it carefully. VAS of Sjogren’s syndrome patients shows the lowest reactivity of the treatment. Thus, thorough history taking of medical records must be required for successful treatment outcomes.

After the treatment phase, the disease has been aggravated among 17 patients. Seven of them had no disease. Two patients had osteoporosis, and the other 4 patients had a history of radiotherapy for cancer. The other 2 patients had psychopathy and take anticonvulsants routinely. This fact indicates that medical comorbidity can affect the results of the treatment phase.

In the case of the successful care groups that marked VAS value under 2.0, 14/41 patients had no medical history. Seven of the 41 patients had thyroid diseases, and 6 of them were taken radiotherapy for treating thyroid cancer. Eighteen of 41 patients were polydrug users. Considering the worst treatment outcome of Sjogren’s syndrome patients, successful cure of these patients is a very difficult and complicated procedure. In this group, a comprehensive treatment plan should be conducted case by case.

Our treatment protocol for systematic treatment according to the patient’s comorbidity is as follows.

### First visit

At the 1st visit, we diagnose the disease thoroughly. Factors that induce hyposalivation can be considered to be various drug doses, aging, facial radiation, chemotherapy, and autoimmune diseases. These factors cause an irreversible change of major salivary gland cells, leading to chronic sialadenitis. Therefore, the patient’s medical history should be investigated thoroughly, the patient’s subjective discomfort should be confirmed by the VAS value, and the unstimulated whole saliva should be measured [7]. The medical history of the patient is very important for diagnosis. And then, an intraoral examination is executed. Stensen’s duct and Wharton’s duct orifice can be detected through an oral examination. With additional stimulation of squeezing from the extraoral area, intraoral salivation can be detected. With this method and UWS, we can compare indirectly the salivation status of patients. In addition, a salivary buffer test can be performed using a pH meter instrument (HANNA instruments HI8424 pH meter), indicating the ability of saliva. If the saliva is acidic, the risk of periodontal disease and dental caries rises. This information can help clinicians in diagnosing hyposalivation more thoroughly.

We are strongly emphasizing the importance of patient education. First, the patient should take enough water and uncooked vegetables. This can make the oral environment sufficiently humid. And they also should refrain from smoking, alcohol, and caffeine. Second, we teach patients tongue mobilization. This can stimulate the minor salivary gland under the tongue to secrete enough saliva. And finally, we teach extraoral oral massage which major salivary gland — especially the submandibular gland—to secure enough secretion of saliva. These methods can alleviate bad symptoms of hyposalivation even in the home. We emphasize these provisions in every visit of the patient in our clinic.

### Second visit

At the 2nd visit, we compile radiographic images of the patient. We take a radiocontrast-enhanced neck CT image (Fig. 1). This image provides us with information about the anatomical structure of the major salivary gland of the patient. With this information, we can diagnose hypertrophy or atrophy of the major salivary gland, especially the parotid and submandibular glands. And if there were sialolithiasis existing, we can easily find it through the CT image. This can help us make an accurate diagnosis. Second, we routinely take scintigraphy of patients (Fig. 2) [8]. This image uses radioactive material, measuring the clearance of the salivary gland. With this material, we can take a quantitative analysis of major salivary gland functions. And we can also compare the salivation of salivary glands. If there were obstacles which can affect the partial or total salivation, decreased salivation can be found in this image. This method is significantly important for us, evaluating the successfulness of treatment and the status of hyposalivation.

### Third visit

At the 3rd visit, treatment will be performed. We can try sialendoscopy, which is a surgical approach and drug therapy. Sialoendoscopy is conducted in the operation room with local anesthesia, using special instruments through major salivary gland ducts including Stensen’s and Wharton’s ducts [13]. At first, we try gradual enlargement of the salivary duct from the #0000 probe. This enlargement extent depends on the severity of ductal stenosis, and the operator should be aware of the duct which is very vulnerable from perforation. Second, we try in a sialendoscope through salivary ducts [14]. In this phase, we can image intraductal status visually and record video, which can reveal the intraductal inflammation status such as obstructed by mucous plugs (Fig. 3). This video record can help the patient understand the procedures and the status of the patient objectively. After this, saline irrigation through the irrigation channel of the sialendoscope is performed. Routinely, 30ml of saline is used, and diluted dexamethasone solution (dexamethasone 5mg/saline 45ml) is used [15]. This irrigational phase is somewhat painful procedure, we use pre-operative NSAID injections for pain control and monitor vital signs of patient. After the procedure, we educate patient about post-operation cautions. Temporary dryness of mouth can occur just after the operation. And routinely day-to-day swelling of extraoral operation site can occur due to irrigational phase. This phenomenon can last 2~3 days, so we need to notice patients about this information.

At the same time, drug therapy is followed. The first choice drug is pilocarpine (Salagen, Eisaikorea), which is a parasympathetic nerve agonist. This drug enhances salivary flow, but can cause systemic hyperhidrosis. The target dosage is 0.5 tablet, twice a day. But it should be modulated depending on severity. If a patient suffers from Sjogren’s disease or thyroid cancer, this drug is within health insurance coverage. And Mu-terasil (Nanosigma biotech) is a complementary choice. This is an intraoral coating agent, which helps intraoral barrier formation.

### Follow-up visits

Three days after the procedure, we take a routine recall check-up. In follow-up visits, we conduct UWS measurement and salivary buffer test. And we compare the data before and after the procedure. Comparing the treatment efficacy found determines whether a further surgical approach will be required. In that time, we cared for the bad symptoms of the patient regarding hyposalivation. This caring approach can alleviate the discomfort of the patient, even if the treatment period is longer than a year. As usual, a follow-up visit is performed 1 month, 3months, and 6 months after the procedure.

In our clinic, we have been treating the patient followed by this protocol. More accumulated data and statistical analysis are needed for accurate diagnosis and projections. In terms of drug administration for those who take drugs routinely, a complex interaction between the patient and the drug can occur which affects salivary gland function. Further study should be completed to find the mechanism of the drug’s action.

## Conclusion

We manage hyposalivation patients with an approach regarding cure and care concept. This concept includes diagnosis, drug therapy, and surgical procedures. Daily discomfort can be alleviated with cooperation between the clinician and the patient. In the case of patients with systemic disease, reactivity of treatment can be reduced. Although it cannot consider all factors that cause hyposalivation, in this study of 154 patients, the discomfort of them after sialendoscopy was reduced. Hyposalivation is a curable and manageable disease in some cases, so interpretation between the clinician and the patient is important.

Currently, many studies on the treatment of hyposalivation with sialendoscopy are in progress. However, a definitive study on the causes of hyposalivation is lacking. Similar to the specific antibodies and proteins detected in autoimmune diseases such as Sjogren’s syndrome, it can be expected that specific proteins will be detected in the major or minor salivary glands in hyposalivation patients. We will not only present a treatment protocol for hyposalivation patients, but also consider methods for diagnosing more precisely and improving treatment efficacy. This study shows encouraging results and invites further studies.

## Data Availability

The datasets used and/or analyzed during the current study are available from the corresponding author on reasonable request.

## Abbreviations

VAS: Visual analogue scale
UWS: Unstimulated whole saliva
CT: Computed tomography
